# Quality Control Criteria for Analysis of Organic Traces in Water

**DOI:** 10.1100/tsw.2002.208

**Published:** 2002-04-18

**Authors:** José L. Martinez Vidal, Antonia Garrido Frenich, Francisco J. Egea González

**Affiliations:** Department of Analytical Chemistry, University of Almería, 04071 Almería, Spain

**Keywords:** quality control criteria, microcontaminants, water, identification, confirmation

## Abstract

The aim of this paper is the discussion of quality control (QC) criteria for environmental monitoring of organic contaminants at trace levels in water. In addition, QC criteria in the identification and confirmation of target analytes have been considered.

## INTRODUCTION

The difficulty of analysing organic microcontaminants in water samples is well known. This is due, among other reasons, to (1) the complexity of the matrix, (2) the wide range of the analytes to be monitorized, including parent compounds, metabolites, and transformation products, and (3) the low concentration levels at the limit of the capabilities of the analytical methods used. In addition, there is a permanent need for quality analytical data about contaminants at trace levels in environmental matrixes to be used in determining compliance with national or international regulations.

Regulation means setting limits. Verifying the compliance with these limits, means chemical measurements. Laboratories of analysis and service companies that provide information must contribute to assuring the quality of their data. In order to reach this aim, the introduction of Quality Assurance (QA) Systems in analytical laboratories is being demanded by customers, because without such implementation it is not possible to assure the quality of the analytical results. In practical terms, QA refers to the overall measures taken by the laboratory to ensure and regulate quality. Such measures include: (1) using traceable methods[1], (2) applying Quality Control (QC) criteria[2,3], (3) participating in proficiency testing[4,5], and (4) getting accreditation[6] according to an international standard, such as ISO 17025[7].

QC describes the measures related to the monitoring and control of the analytical data. QC is introduced in the laboratory to optimise sampling, as well as all steps of the measure-chemical process (MCP), and the treatment of analytical signals, and to assure that the analytical data obtained from the application of an analytical method are under statistical control[2].

Currently, the use of QC measures should be recommended before publishing environmental analytical data. Such measurements involve the use of blanks, duplicated samples, certified reference materials (CRM), blind samples, and standards or spiked samples, among others. These QC measures, together with documented criteria related to the identification and confirmation of the identity of the target analytes, should be considered as indications of drift in the system.

## QC CRITERIA FOR ANALYSIS OF ORGANIC TRACES IN WATER

The frequency of QC measures will depend on the volume of work, the experience of the analysts, costs involved, and the risk that laboratory management will take to assure that the analytical system is under control. However, certain operations of QC must be carried out in a routine way.

QC criteria must be applied after analysing a certain number of samples. An important step is to establish the right size of the set. For a multiresidue chromatographic method with total analysis time of about 60 minutes, an adequate size of the set may be about 12 samples, which, together with the analysis of blanks, duplicated and blind samples, CRM, calibration standards, and spiked samples, as well as the injection of one or two aliquots of solvent in order to clean the equipment after the set, can complete a time of about 24 hours.

In any case, it is necessary to analyse the scientific data, in order to document the right size of the set, so as to avoid affecting the analytical results.

### Quality Criteria in the MCP

**Blanks** — The use of various types of blanks[8], such as laboratory reagent blanks and field reagent blanks, allows the analyst to ensure that the analytical signal is not affected by contamination and that it is attributable just to the analyte[9,10]. Reagents, solvents, and water, as well as volumetric equipment, containers, instruments, and laboratory environment can frequently be sources of contamination and must therefore be checked for it (Fig. 1).

**CRM or quality sample** — CRM can be used to study the variation between sets of a particular analysis. Reference materials for organic traces in waters may be supplied using a reference

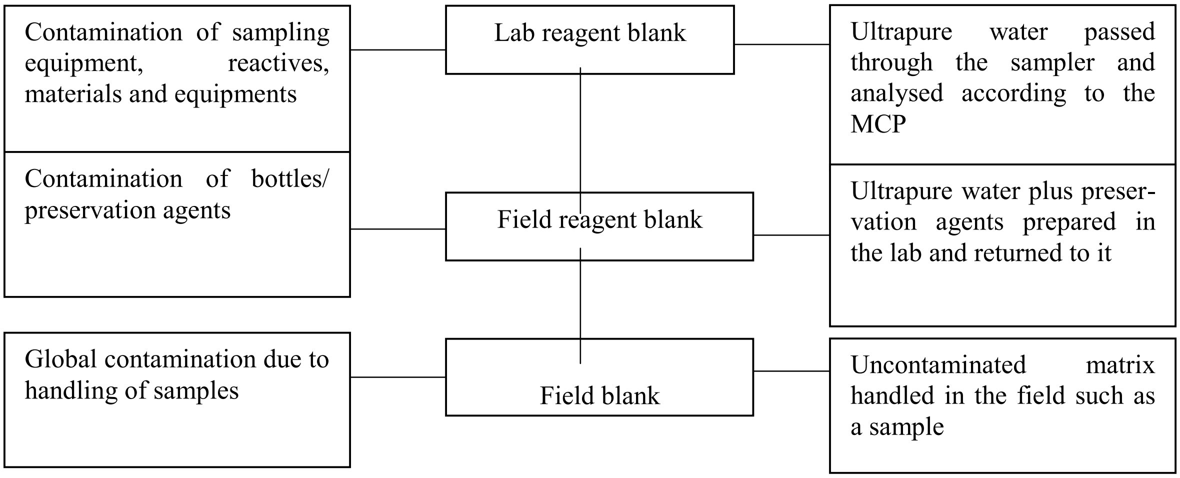

**FIGURE 1.** Types of blanks used in the analysis of organic traces in water samples.

material made at home, spiking a sample of water with 5 to 10% of the target analytes eluting along the chromatogram. This sample, properly stored, must be checked for stability and homogeneity and then used as a quality sample.

**Duplicated and blind samples** — Duplicated samples provide a less formal means than quality samples for checking for drift. Normally, the duplicated sample is a conventional sample, repeated later on in the batch of samples or perhaps in a different batch. Duplicated samples are located in the set every certain number of samples (1 every 10 samples). Blind samples are repeated samples placed in the analytical bath without the knowledge of the analyst. Blind samples are complementary with duplicated samples, giving us information about analyst proficiency.

**Standards** — A chemical measurement system presents two sides of calibration, i.e., instrumental and methodological calibration[11]. The last one involves the use of chemical standards, which may be measured separately from samples (external standards) or as part of the samples (internal standards). Calibration plots assure the proper use of the analytical instrumentation. The slopes of the daily calibration graphs must not differ significantly from the value found in the validation of the method. A decrease in the slope value may be critical when we are working with values close to the quantitation limit. Matrix influence[12,13] on the calibration curve also has to be considered.

**Laboratory/Field spiked samples** — Field spiked samples, prepared at the sampling site, give us information about losses of analytes due to adsorption, volatilisation, or degradation processes. Recoveries carried out with each set of samples can be used as a way to check that the analytical method is under statistical control and that the variability of the results are included in the range established by the uncertainty of the method.

An analyte surrogate can be added to all the samples before starting the analysis, in order to demonstrate that the MCP has been satisfactory in each sample and that mistakes have been avoided, i.e., to monitor method performance with each sample.

## Quality Criteria in the Identification and Confirmation of Target Analytes

**Identification** — Identification criteria set a retention time window (RTW) based on the reference retention time within which the analyte peak for a real sample must occur[14].

**Confirmation** — Several alternatives may be used in chromatographic techniques: a chromatographic column of significantly different polarity, a detector operating on a different principle, or gas chromatography to complement liquid chromatography. Another alternative may be by simultaneous acquisition of combined chromatographic and spectral data (UV/VIS, fluorescence, atomic-emission, or especially mass spectrometry).

**Confirmation by mass spectrometry** — The confirmation under selected ion monitoring (SIM) conditions requires that the inter-ion abundance ratios of at least two, or preferably three, noncontiguous ions be within ±10 to 20% of the absolute relative abundance obtained for a reference standard of the compound analysed under the same conditions.

In case of using MS/MS spectra, the identity is confirmed if the intensity ratios for the main ions are within approximately 20% of those obtained on the same day from the reference standard, or the spectral fit obtained comparing the obtained spectrum with the reference standard (stored in an appropriate library) must to be ≥700. In addition, a signal-to-noise ratio of at least 3:1 (10:1) from a chromatographic peak is required for qualitative (quantitative) purposes.

## CONCLUSIONS

Currently, the analytical information requested must be delivered with increasing quality statements. This is an important topic, since chemical measurements support important decisions in various fields as well as for the mutual acceptance of data, both nationally and internationally. Obviously, this topic has great relevance in the analysis of organic traces in a wide range of environmental matrixes. In consequence, QC criteria must be applied in the chemical laboratories in order assure to their adaptation to this new but increasingly widespread situation.

## ACKNOWLEDGEMENTS

The authors are grateful to Spanish DGCICYT (project PB97-0789-C02-02) for financial support. This paper was presented at the CSIC/ESF workshop Analysis, Toxicity and Biodegradation of Organic Pollutants in Groundwater from Contaminated Land, Landfills and Sediments, Barcelona, Spain, 8–10 November 2001.
